# Improving Cyclability of All‐Solid‐State Batteries via Stabilized Electrolyte–Electrode Interface with Additive in Poly(propylene carbonate) Based Solid Electrolyte

**DOI:** 10.1002/advs.202105448

**Published:** 2022-03-03

**Authors:** Pravin N. Didwal, Rakesh Verma, An‐Giang Nguyen, H. V. Ramasamy, Gwi‐Hak Lee, Chan‐Jin Park

**Affiliations:** ^1^ Department of Materials Science and Engineering Chonnam National University 77, Yongbong‐ro, Buk‐gu Gwangju 61186 South Korea; ^2^ Department of Materials University of Oxford Parks Road Oxford OX1 3PH UK; ^3^ Davidson School of Chemical Engineering Pardue University West Lafayette IN 47907 USA

**Keywords:** all‐solid‐state lithium‐ion batteries, interfacial stability, solid polymer electrolytes, tetraethylene glycol dimethyl ether additive, tissue membrane

## Abstract

In this study, tetraethylene glycol dimethyl ether (TEGDME) is demonstrated as an effective additive in poly(propylene carbonate) (PPC) polymers for the enhancement of ionic conductivity and interfacial stability and a tissue membrane is used as a backbone to maintain the mechanical strength of the solid polymer electrolytes (SPEs). TEGDME in the PPC allows the uniform distribution of conductive LiF species throughout the cathode electrolyte interface (CEI) layer which plays a critically important role in the formation of a stable and efficient CEI. In addition, the high modulus of SPEs suppresses the formation of a protrusion‐type CEI on the cathode. The SPE with the optimized TEGDME content exhibits a high ionic conductivity of 0.89 mS cm^−1^, an adequate potential stability of up to 4.89 V, and a high Li‐ion transference number of 0.81 at 60 °C. Moreover, the Li/SPE/Li cell demonstrates excellent cycling stability for 1650 h, and the Li/SPE/LFP full cell exhibits an initial reversible capacity of 103 mAh g^−1^ and improved stability over 500 cycles at a rate of 1 C. The TEGDME additive improves the electrochemical properties of the SPEs and promotes the creation of a stable interface, which is crucial for ASSLIBs.

## Graphical abstract

The effects of adding tetraethylene glycol dimethyl ether to poly(propylene carbonate)‐based solid polymer electrolyte (SPE) having tissue paper backbone are demonstrated. The all‐solid‐state battery (ASSB) with optimized SPE exhibits better electrochemical performance and forms a stable cathode–electrolyte interface (CEI) on LiFePO_4_. The LiF layer plays a critically important role in the fabrication of a stable and efficient CEI insulator and is believed to provide a stable interface and higher stability of the cathode, leading to excellent cyclability of ASSB.

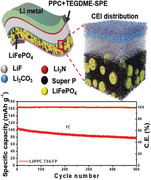

## 1 Introduction

Li‐ion batteries (LIBs) are among the most promising scalable energy storage devices in the industrial market owing to their high energy density, long cycle life, and limited self‐discharge.^[^ ^1^, ^2^, ^3^ ^]^ Despite these advantages, LIBs have caused catastrophic battery incidents, and thus, their safety is a major concern.^[^ ^4^ ^]^ Although LIBs with a Li metal anode have gained recent attention, the interfacial chemical and electrochemical instability of organic‐liquid electrolytes induces the formation of Li dendrites on Li metal.^[^ ^4^, ^5^ ^]^ Li dendrites can penetrate the separator membrane, leading to short‐circuiting, thermal runaway, and ultimately, fire and accidents. These safety issues can be addressed by replacing flammable organic‐liquid electrolytes with solid‐state electrolytes.^[^ ^6^, ^7^ ^]^ Although the current developments of solid‐state electrolytes are appealing, the limitations associated with the interfacial properties of electrodes have not been thoroughly investigated.

Solid polymer electrolytes (SPEs) offer significant advantages compared to organic‐liquid electrolytes, such as no leakage, non‐flammability, wide electrochemical potential window, and ease of fabrication.^[^ ^8^, ^9^ ^]^ In addition, SPEs exhibit superior interfacial contact with electrodes compared to ceramic electrolytes.^[^ ^10^ ^]^ However, the low ionic conductivity, poor mechanical strength, and chemical instability of SPEs are the main challenges that limit the overall electrochemical performance of all‐solid‐state Li‐ion batteries (ASSLIBs).^[^ ^11^, ^12^ ^]^ Therefore, various approaches have been developed to enhance the ionic conductivity and mechanical strength of SPEs. To date, the incorporation of ceramic fillers, cross‐linking of polymers, addition of liquid plasticizer, introduction of two or more Li salts, and use of a host membrane or backbone are the most commonly adopted approaches.^[^ ^13^, ^14^, ^15^, ^16^ ^]^ Although several studies have found that adding ceramic fillers to SPEs is one of the most straightforward ways to improve ionic conductivity and mechanical strength, optimizing the concentration, size, shape, and passive nature of the filler can indirectly affect the energy density and cost of ASSLIBs.^[^ ^17^, ^18^, ^19^ ^]^ Moreover, SPEs form an interphase when they react with Li metal and electrodes, which is difficult to control and leads to a non‐uniform, unstable interlayer between electrolytes and electrodes.^[^ ^20^ ^]^ The uncontrollable growth of an ionic non‐conducting or insulating interlayer increases the total resistance, causes poor interfacial contact with electrodes, and reduces the safety and electrochemical performance of the battery.^[^ ^21^, ^22^, ^23^, ^24^ ^]^ Therefore, it is essential to develop a facile approach to control the electrochemical properties of SPEs in order to obtain a stable interface, high energy density, and low cost for preparing ASSLIBs.

The introduction of an organic‐liquid additive into SPEs is a potential approach for significantly enhancing the electrochemical properties, resulting in high energy density and low cost.^[^ ^25^, ^26^, ^27^, ^28^, ^29^ ^]^ Ethylene carbonate, polyethylene glycol dimethyl ether, propylene carbonate, and succinonitrile have been successfully used as additives to increase ionic conductivity by an order of magnitude.^[^ ^9^, ^30^, ^31^, ^32^, ^33^ ^]^ Even a low concentration of additive can plasticize the polymer chain, enhance the rate of salt dissociation, increase the amorphous nature of the polymer, and boost the mobility of Li ions.^[^ ^25^, ^26^, ^27^, ^28^, ^29^ ^]^ Moreover, the incorporation of an additive into a polymer system opens pathways for Li‐ion transportation through the plasticizer‐modified interface.^[^ ^34^ ^]^ The additive increases the amorphous regions in the polymer and decreases the degree of crystallinity of SPEs. The highly amorphous nature of SPEs can reduce the energy barrier for ionic movement and facilitate fast Li‐ion transport.^[^ ^25^, ^26^ ^]^ Therefore, the combination of an ionic conducting polymer and an additive is a highly promising solution for satisfying the requirements of LIBs. Moreover, electrolyte additives have been investigated in order to achieve significant innovation in the cyclability of LIBs without forgoing energy density. This was accomplished by creating stable electrode interfacial structures and removing reactive chemicals.^[^ ^32^, ^33^ ^]^ Therefore, the development of electrolyte additives has been targeted as a means to obtain a stable and robust interphase on electrodes.^[^ ^33^ ^]^ To mitigate the aforementioned concerns and improve the interfacial stability of SPEs with electrodes, it is essential to understand the origin of the stable interphases.

In this investigation, we designed and engineered SPEs with excellent total ionic conductivity, high enough mechanical strength, and high electrochemical stability with Li metal and a LiFePO_4_ (LFP) cathode. We introduced tetraethylene glycol dimethyl ether (TEGDME) as an additive in a poly(propylene carbonate) (PPC) polymer to improve the ionic conductivity and modify the electrode–electrolyte interphase. The mechanical strength of the SPE was also maintained by using a tissue membrane as a backbone. In addition, density functional theory (DFT) calculations were employed to study the effect of the additive on the electrochemical potential stability of SPE. The SPE with an appropriate concentration of TEGDME demonstrated a high total ionic conductivity of 0.89 mS cm^−1^, a large enough Li‐ion transference number of 0.81, and superior mechanical strength of 6.9 MPa. The Li/Li symmetric cell exhibited excellent stability of up to 1650 h, and the Li/LFP cell had a high initial discharge capacity of 103 mAh g^−1^ and retention of 77% after 500 cycles at a rate of 1 C at 60 ℃. The high dielectric constant and low molecular weight of TEGDME not only increased the ionic conductivity of SPE but also resulted in the formation of a stable interface with Li metal and the LFP cathode. These results could contribute toward the development of simple, cost‐effective ASSLIBs with excellent electrochemical performance.

## 2 Results and Discussion

**Scheme 1:**
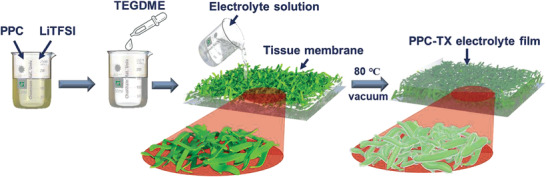
Conceptual design of the fabrication of PPC‐based composite solid polymer electrolyte film.

The SPE film was fabricated by a simple casting method as shown in **Scheme** **1**. The SPE consisted of various contents of the TEGDME additive in polypropylene carbonate+lithium bis(trifluoromethanesulfonyl)imide salt (PPC+LiTFSI). Photographs of a typical PPC‐T5 (5 wt% of TEGDME) SPE are presented in Figure S1, Supporting Information. The fabricated PPC‐T5 film was highly flexible and semi‐transparent, suggesting that this film could be used in flexible batteries.

### 2.1 Microstructural, Structural, and Thermal Analyses of SPEs

**Figure 1:**
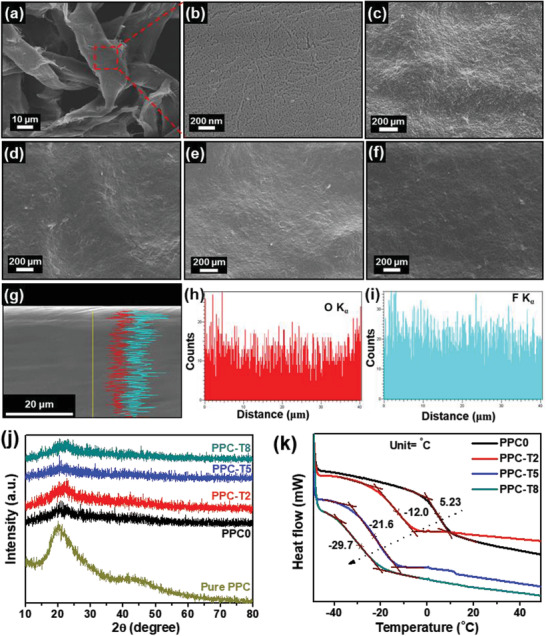
a,b) Top‐surface SEM images of tissue paper at low and high magnification. Top‐view SEM images of c) PPC0, d) PPC‐T2, e) PPC‐T5, and f) PPC‐T8. g) Cross sectional SEM image of PPC‐T5 and h,i) corresponding EDS line elemental mapping. j) XRD pattern and k) DSC curve of PPC0, PPC‐T2, PPC‐T5, and PPC‐T8.

Scanning electron microscopy (SEM) imaging was performed to observe the surface and cross‐section of the PPC0 without TEGDME additive and PPC‐TX (X = 2–8) films in which 0–8 wt% TEGDME was included. In addition, the corresponding energy‐dispersive X‐ray spectroscopy (EDS) elemental mapping was obtained to observe the distribution of LiTFSI salt within the fabricated PPC‐TX film. **Figure** **1**a,b shows the SEM images of the tissue membrane that was used as the backbone of the SPEs. The tissue membrane exhibited micropores that were formed due to the arbitrary alignment of the fibers, which contained nano‐pores. Furthermore, the top‐view and side‐view morphologies of PPC0, PPC‐T2, PPC‐T5, and PPC‐T8 are shown in Figure 1c–f and Figure S2, Supporting Information, respectively. All the PPC‐TX films contained void‐free top and side surfaces. The smoothness of the surface increased with the addition of the TEGDME additive. Thus, the incorporation of the additive into the PPC‐TX resulted in improved surface smoothness, which can further enhance the interfacial contact between the electrolyte and the electrode. To investigate the distribution of PPC and LiTFSI salt in the PPC‐X film, the SEM–EDS elemental line mapping of PPC‐T5 was conducted (Figure 1g–i) and SEM–EDS spectrum with elemental quantitative analysis is shown in Figure S3, Supporting Information. Interestingly, the results of the EDS line mapping revealed that the O element in the PPC and the F element from the LiTFSI were consistently spread throughout the film. This indicated that the PPC and LiTFSI salt were well distributed in the PPC‐T5 films.

Structural analysis of PPC‐TX films was performed using X‐ray diffraction (XRD). The XRD patterns of PPC‐TX for various TEGDME additive contents are shown in Figure 1j. A broad peak was observed at 2*θ* ≈ 20°, confirming the low crystallinity of PPC.^[^ ^37^ ^]^ Comparative difference in the relative intensity of the XRD peak was observed after the introduction of the TEGDME additive into the PPC. The relative intensity of the XRD peak at 2*θ* ≈ 20° of the PPC‐TX SPE was lower than that of the original PPC because the amorphous nature and the degree of disorder of the PPC‐TX SPE film slightly increased with the addition of TEGDME.^[^ ^38^, ^39^ ^]^ The thermal stability of the SPE is important in terms of battery safety when it comes to high temperature. Thus, the thermal stabilities of the PPC‐TX films were determined for various contents of the TEGDME additive using thermogravimetric analysis (TGA) (Figure S4, Supporting Information). Weight loss was observed for all PPC‐TX films above 220 ℃. This indicated that the fabricated PPC‐TX was thermally stable up to 220 ℃ in an inert atmosphere. Differential scanning calorimetry (DSC) was used to investigate the influence of the TEGDME additive on the thermal reversibility and glass transition temperature (*T_g_*) of the SPEs. Figure S5, Supporting Information, shows the reversible behavior of the PPC‐TX during heating and cooling, which implies outstanding thermal reversibility. The PPC0, PPC‐T2, PPC‐T5, and PPC‐T8 films exhibited *T_g_* values of 5.23, −12.0, −21.6, and −29.7 ℃, respectively (Figure 1k). Owing to the interaction between PPC and TEGDME, the TEGDME could effectively act as a plasticizer in PPC, causing a significant reduction in the *T_g_* of PPC‐TX containing the TEGDME additive. The smaller *T_g_* values indicate the greater mobility of the PPC polymer chain.^[^ ^11^ ^]^ This implied that, because of the increase in amorphous regions, the mobility of the PPC‐TX film increased significantly with increasing content of the TEGDME additive.^[^ ^38^ ^]^

### 2.2 Interaction between PPC and TEGDME

**Figure 2:**
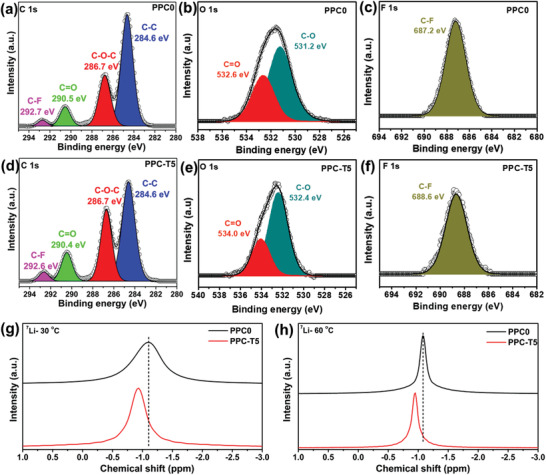
a) C 1s, b) O 1s, and c) F 1s core XPS spectra of PPC0; d) C 1s, e) O 1s, and f) F 1s core XPS spectra of PPC‐T5. Solid‐state ^7^Li MAS‐NMR spectra of PPC0 and PPC‐T5 at g) 30 and h) 60 °C.

The interactions of the functional group of PPC with the TEGDME additive and LiTFSI salt were investigated via X‐ray photoelectron spectroscopy (XPS). The high‐resolution C 1s, O 1s, and F 1s peaks for PPC0 and PPC‐T5 were deconvoluted as shown in **Figure** **2**a–f. The interaction between PPC polymer and LiTFSI salt was evident due to the presence of C–F sub‐peak at a binding energy of ≈292.7 eV in the C 1s spectrum for both cases.^[^ ^41^ ^]^ This was further supported by the presence of F 1s peak at binding energy of ≈687.2 eV. The LiTFSI salt and PPC interact through the electronegative F atom of LiTFSI and electropositive carbonyl carbon (C═O) of PPC. In addition, the interaction of the PPC polymer with the TEGDME additive was established by analyzing the C 1s and O 1s peaks. According to the conventional chemistry, it is understandable that TEDGME interact with PPC through its electron‐rich O functional group (CH_3_—O—) and the electro‐positive carbonyl carbon (C═O) of PPC. Interestingly, the deconvoluted O 1s peak shifted to a higher binding energy for PPC‐T5 compared to PPC0. Such observations clarify the interaction of PPC with TEDGME through O functionality. Moreover, the peak shifting confirms that TEDGME interacts more closely with PPC than with LiTFSI. To further confirm the same phenomenon, high‐resolution C 1s and O 1s peaks were analyzed for PPC+TEDGME without LiTFSI and similar changes were observed in O 1s peaks (Figure S6, Supporting Information). However, in this case, the peak shift for the O 1s sub‐peak was smaller compared to PPC‐T5, which was evident due to the absence of LiTFSI. Moreover, a clear splitting of the O1s peak was shown to further confirm the interaction of PPC with TEDGME. Based on the above discussion, it is now clear that PPC and TEDGME chemically interact through the O functionality of TEDGME and the carbonyl carbon of PPC (C═O). Moreover, the coordination of Li with PPC after the addition of TEGDME was determined via ^7^Li magic‐angle‐spinning (MAS) solid‐state nuclear magnetic resonance (NMR) spectroscopy at 30 and 60 °C. Figures 2g and h show the ^7^Li MAS‐NMR spectra of PPC0 and PPC‐T5 at 30 and at 60 °C, respectively. The NMR peak of ^7^Li in PPC‐T5 was shifted upfield at both temperatures. Moreover, the linewidth of the ^7^Li peak was narrower for PPC‐T5, as compared to PPC0. The Li‐ion coordination was primarily responsible for the upfield shifting of the ^7^Li peak after the addition of TEGDME. The TEGDME content in the PPC changed the local environment of Li and resulted in a strong interaction with TEGDME. The slight reduction in the linewidth and the upfield shifting of the spectra were indicative of mobility augmentation and rapid ion diffusion in the PPC‐T5. In summary, the results of the XPS and MAS‐NMR studies confirmed the interaction of the PPC polymer with the TEGDME additive and the Li‐ion coordination between them.

### 2.3 Electrochemical Analysis of SPEs

**Figure 3:**
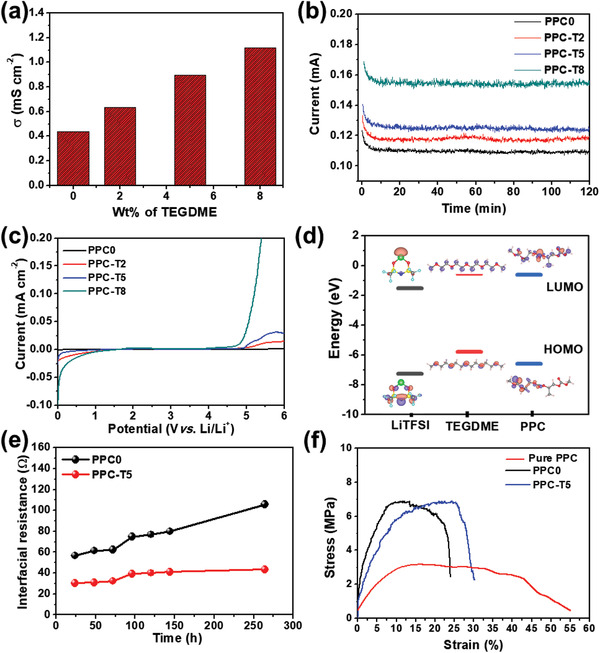
a) Ionic conductivity of CSPE as a function of the TEGDME content at 60 °C. b) Current transient curves of PPC0, PPC‐T2, PPC‐T5, and PPC‐T8 at 60 °C. c) Linear sweep voltammograms of PPC0, PPC‐T2, PPC‐T5, and PPC‐T8 at 60 °C. d) HOMO and LUMO energy levels of PPC, LiTFSI, and TEGDME obtained via DFT calculations. e) Plot of interfacial resistance of Li/Li symmetric cell with PPC0 and PPC‐T5 as a function of storage time at 60 °C. f) Stress–strain curves of pure PPC, PPC0, and PPC‐T5.

**Scheme 2:**
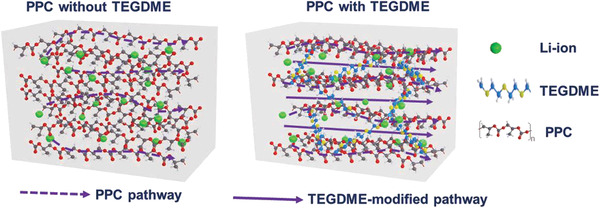
Schematic of the Li‐ion transportation phenomenon in the PPC polymer electrolyte film with and without TEGDME.

The effect of the TEGDME additive on the ionic conductivity (*σ*) of the PPC‐TX was evaluated based on electrochemical impedance spectroscopy (EIS) measurements, and the corresponding histograms are shown in **Figure** **3**a. The original Nyquist plots of PPC0, PPC‐T2, PPC‐T5, and PPC‐T8 at 60 ℃ are shown in Figure S7, Supporting Information. PPC0, PPC‐T2, PPC‐T5, and PPC‐T8 had *σ* values of 0.43, 0.63, 0.89, and 1.12 mS cm^−1^, respectively. With an increase in the content of the TEGDME additive, these values increased, which could be attributed to the increase in amorphous regions and decrease in the *T_g_*. The Li‐ion transportation phenomenon in the electrolyte film with and without the TEGDME additive in the PPC polymer is explained schematically (**Scheme** **2**). The mobility and transport pathways of the Li ions through the electrolyte can affect the conductivity of the SPEs.^[^ ^40^, ^41^ ^]^ In the case of the PPC0 electrolyte, the Li‐ion can only be transported through the PPC polymer cross‐links, resulting in limited ionic conductivity. The addition of the TEGDME additive to the PPC polymer created a superfluous pathway for Li‐ion conduction via the TEGDME‐modified PPC polymer interface.^[^ ^42^, ^43^, ^44^ ^]^ The Li‐ion conduction in the PPC containing TEGDME can occur via two pathways: PPC polymer and TEGDME‐modified PPC interface. The Li‐ions are mainly transported through the TEGDME‐associated polymer interface, which increases the total ionic conductivity of the SPEs. In addition, TEGDME increases the amorphous regions in the PPC polymer, which leads to intensified segmental motion of the polymer chain.^[^ ^42^ ^]^ Moreover, the ionic conductivity of the SPEs is inhibited by ion segregation of the charged species because ion pairs are charged multiples of the ions. Low ionic conductivity can also be observed when a Li salt with large coulombic interactions or high lattice energy is added to the polymer host.^[^ ^43^, ^44^ ^]^ The ion‐dissociation energy of the polymer must be higher than the ion‐pairing stabilization. An additive can plasticize the polymer chain and enhance the dissociation rate of the salt.^[^ ^42^ ^]^ A significant improvement in the ionic conductivity is observed for the PPC containing TEGDME owing to the increase in the proportion of amorphous regions, ion mobility, and re‐establishment of the ion‐conduction channels within the TEGDME‐modified PPC.

Furthermore, the Li‐ion transference number (*t* **_Li+_)** of PPC‐TX was calculated based on chronoamperometry results acquired at 60 ℃ because *t* **_Li+_** is one of the critical parameters that define an efficient SPE. The current transition curves of PPC0, PPC‐T2, PPC‐T5, and PPC‐T8 and its Nyquist plots before and after polarization are presented in Figure 3b and Figure S8, Supporting Information, respectively. PPC0, PPC‐T2, PPC‐T5, and PPC‐T8 delivered *t* **_Li+_** of ≈0.76, ≈0.79, ≈0.81, and ≈0.84, respectively. The interaction of the TEGDME additive and the PPC polymer chain increased the size of the amorphous areas, forming a plasticizer‐modified phase enabling Li‐ion transit through the electrolyte as *t* **_Li+_** raised (explained in Scheme 2). Therefore, the TEGDME additive boosted the Li‐ion mobility and facilitated further improvement in the battery performance.

Although the ionic conductivity and *t* ***_Li+_*** of SPEs play a vital part in enhancement of ASSLIBs performance, the potential stability of SPEs is a crucial parameter in choosing a potential window for the cell. The effect of the TEGDME additive content on the electrochemical potential stability of PPC was determined via linear sweep voltammetry (LSV). The LSV curves of PPC0, PPC‐T2, PPC‐T5, and PPC‐T8 with an applied scan rate of 1 mV s^−1^ at 60 ℃ are presented in Figure 3c. The LSV curves of PPC0, PPC‐T2, PPC‐T5, and PPC‐T8 showed anodic breakdown at ≈5.25, ≈5.01, ≈4.89, and ≈4.52 V, respectively, versus Li/Li^+^. As the TEGDME additive content increased, the decomposition potential shifted to the lower‐potential side. However, there is limited information regarding the reduction in the electrochemical potential stability with an increase in the TEGDME additive content in SPEs. Therefore, we theoretically investigated this phenomenon using the DFT method. In parallel with the LSV test, we calculated the highest occupied molecular orbital (HOMO) and the lowest unoccupied molecular orbital (LUMO) energy levels of LiTFSI, TEGDME, and PPC, and correlated these values with the electrochemical potential window. Based on thermodynamic analyses, the HOMO level represents the electrochemical oxidation potential of an electrolyte. Therefore, the electrolyte with a high HOMO energy level has a lower oxidation potential stability.^[^ ^43^ ^]^ Specifically, the HOMO and lowest unoccupied molecular orbital (LUMO) levels of the LiTFSI, TEGDME, and PPC are shown in Figure 3d. The HOMO energy of TEGDME was −5.79 eV, which was higher than those of LiTFSI (−7.26 eV) and PPC (−6.60 eV). Thus, it is expected that the anodic oxidation of TEGDME commences prior to that of LiTFSI and PPC. Based on the DFT investigation, the addition of TEGDME in the PPC+LiTFSI SPEs shifted the anodic oxidation to the lower‐potential region. The DFT calculations supported the experimental LSV data in this study.

Although the PPC‐T8 exhibited better total ionic conductivity (*σ* = 1.12 mS cm^−1^) and a higher *t* **_Li+_** (0.84), the electrochemical potential stability was lower than that of PPC‐T5. Nevertheless, PPC‐T5 had a notable ionic conductivity (*σ* = 0.89 mS cm^−1^), adequately high *t* **_Li+_** (0.81), superior electrochemical potential stability (4.89 V versus Li/Li^+^), and outstanding interfacial resistance stability with Li metal. Therefore, PPC‐T5 was selected as the superior candidate for further analysis of the efficient SPE. The most important parameter is the interfacial resistance (*R*_i_) between the Li metal and the SPE, which needs to be reduced for the efficient ASSLIBs. *R*_i_ increases with time, leading to high polarization and rapid degradation during cycling. Therefore, the *R*i between PPC‐TX and Li metal was tested for 24–264 h at 60 °C as a function of ageing time. Plots of *R*_i_ versus ageing time and corresponding Nyquist plots are shown in Figure 3e and Figure S7, Supporting Information, respectively. The Li/PPC0/Li and Li/PPC‐T5/Li cells possessed *R*_i_ values of 56.7 and 30.5 Ω, respectively, after 24 h of ageing. This indicates that the *R*_i_ between the PPC‐TX films and Li metal decreased with the addition of TEGDME. For PPC0, *R*_i_ increased significantly by 246 h. However, for PPC‐T5, *R*_i_ slightly increased up to 96 h and stabilized up to an aging time of 246 h. The *R*_i_ between Li metal and PPC‐T5 was effectively smaller than that between Li metal and PPC0 after 246 h. Incorporation of TEGDME into PPC‐T5 can improve the surface smoothness, further enhancing the interfacial contact between the electrolyte and the electrode. In addition, in the case of PPC‐T5, it can be observed that *R*_i_ is stabilized over time, which can be attributed to the stable interfacial layer on Li metal. In contrast, in the case of PPC0, *R*_i_ gradually increases with the storage time, which may be attributed to the unstable passive layer on the surface of the Li metal.^[^ ^45^, ^46^, ^47^ ^]^ This indicates that the TEGDME additive increased the smoothness and softness of the SPE film, reduced the interfacial contact resistance, and increased chemical stability, leading to the formation of stable interface layer on the surface of Li metal.

### 2.4 Mechanical Properties of SPEs

Although the mechanical strength of the SPE does not contribute directly to its electrochemical performance, it is helpful for suppressing Li dendrite penetration through the electrolyte membrane and preventing short‐circuiting of the battery.^[^ ^48^ ^]^ The mechanical strengths of the pure PPC, PPC0, and PPC‐T5 were investigated based on tensile tests (Figure 3f). The pure PPC film had a lower mechanical strength (≈3.17 MPa), which is insufficient to prevent Li dendrite penetration and can thus compromise the safety of the battery.^[^ ^48^ ^]^ Moreover, the additive in SPE decreases the mechanical strength, and thus, it is difficult to prevent the penetration of Li dendrites. Considering this aspect, many researchers are developing strategies to maintain the strength of the SPE, including the introduction of a backbone.^[^ ^35^, ^36^ ^]^ In addition, the porous nature of the backbone can be beneficial. Given that a polymer can be inserted into the pores of the backbone, this creates an opportunity to develop a thin electrolyte with a high mechanical strength.^[^ ^35^ ^]^ Therefore, we maintained the mechanical strength of the SPEs by introducing a cost‐effective, lightweight, and highly porous tissue membrane as the backbone (revealed in the SEM in Figure 1a,b). The PPC0 had a tensile strength of ≈7 MPa, which was almost 2.2‐fold higher than that of the pure PPC film. This study revealed that the PPC's mechanical strength improved primarily as a result of the tissue membrane serving as a backbone. The PPC polymer can be filled into the backbone, and the micro‐ and nano‐pores of the tissue membrane can help enhance the mechanical strength of the SPE film. PPC‐T5 also exhibited an impressive tensile strength of ≈6.9 MPa, which was almost equal to the tensile strength of PPC0. This notable improvement in the mechanical performance of PPC‐T films was mainly attributed to the tissue membrane, which acts as a nano/microporous backbone.

### 2.5 Compatibility of PPC‐TX with Li Metal

**Figure 4:**
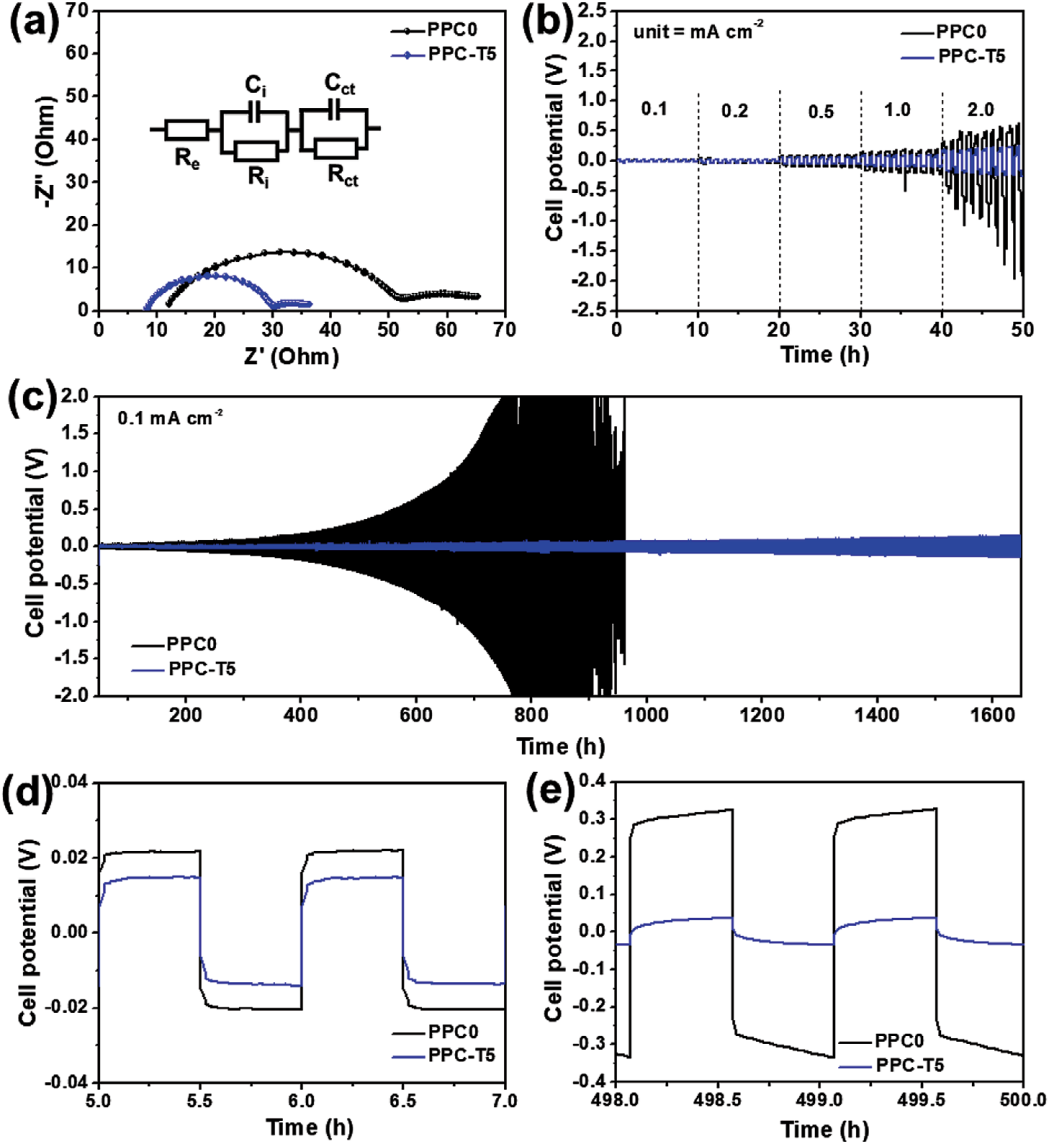
a) Nyquist plot of Li/Li symmetric cells with PPC0 and PPC‐T5 at 60 °C. b) Rate capability of a Li/Li symmetric cell with PPC0 and PPC‐T5 at 60 °C. c) Cyclability of an Li/Li symmetric cell with PPC0 and PPC‐T5 for a current density of 0.1 mA cm^−2^ at 60 °C and d,e) corresponding Li stripping/plating profile at different cycles.

The compatibility between Li metal as an anode with PPC0 and PPC‐T5 during the electrochemical process was analyzed by assembling Li/Li symmetric cells and testing them at 60 °C. First, the *R_i_* of PPC‐TX with Li metal was determined based on EIS of the Li/Li symmetric cell. **Figure** **4**a shows the Nyquist plots of the Li/Li symmetric cells with PPC0 and PPC‐T5 and their corresponding equivalent circuit (inset of Figure 4a). Three types of resistances were included in the Nyquist plot of both the Li/Li symmetric cells: the ohmic resistance at a high frequency, which is associated with the electrolyte resistance (*R_e_*); the interfacial resistance between the Li metal and the PPC‐TX electrolyte (*R_i_*) at a moderate frequency, which is denoted by the semicircle; and the charge‐transfer resistance (*R_ct_*) at lower frequency, which is represented by the depressed semicircle.^[^ ^46^ ^]^ The Li/PPC‐T5/Li cell has a *R_i_* value of 22 Ω, which is lower than that of the Li/PPC0/Li cell (39 Ω). This indicates that the interfacial contact between the SPE and Li metal was significantly improved after the addition of TEGDME to the PPC polymer because the surface of PPC‐T5 softened thereafter.

Furthermore, Figure 4b shows the Li stripping/plating of both the Li/Li symmetric cells at different currents ranging from 0.1 to 2 mA cm^−2^ for each of the 5 cycles (1 cycle = 1 h) at a temperature of 60 °C. The overpotential (*η*) of both the Li/Li symmetric cells with PPC0 and PPC‐T5 increased as a function of the applied current density. The Li/PPC0/Li and Li/PPC‐T5/Li symmetric cells had *η* values of 0.98 and 0.23 V, respectively, at pretty large rate of 2 mA cm^−2^ at 60 °C. The Li/PPC‐T5/Li cell had a fourfold lower *η* value compared to that of the Li/PPC0/Li cell even at a large rate of 2 mA cm^−2^. In addition, the cyclability of the Li/Li symmetric cell with PPC0 and PPC‐T5 was tested at a fixed current density of 0.1 mA cm^−2^ at 60 °C to investigate the stability of SPE with Li metal, as shown in Figure 4c; the corresponding cyclic profiles are shown in Figure 4d,e. The Li/PPC‐T5/Li cell showed an excellent stability of up to 1650 h without cell failure. However, the Li/PPC‐T5/Li cell exhibited large fluctuations with time and failed after 800 h. Moreover, the Li/PPC0/Li and Li/PPC‐T5/Li cells had initial *η* values of 21 and 14 mV, respectively; however, after 500 cycles, the *η* values increased to 332 and 36 mV, respectively, at 0.1 mA cm^−2^. A significant increase in *η* was observed for the Li/PPC0/Li cell after 500 cycles, which indicates the instability of the PPC0 electrolyte with Li metal. By contrast, only a slight change in *η* was observed for the Li/PPC‐T5/Li symmetric cell after 500 cycles. This result also confirms the superior stability of the PPC‐T5 electrolyte with Li metal.

In addition, the stability of PPC‐T5 with Li metal was monitored at 30 °C. Figure S10a, Supporting Information, shows the Nyquist plots of the Li/Li symmetric cells with PPC0 and PPC‐T5 and their corresponding equivalent circuit (inset of Figure S10a, Supporting Information). The Li/PPC‐T5/Li cell had a *R_i_* value of 290 Ω, which is twofold lower than that of the Li/PPC0/Li cell. This indicated that the interfacial contact between the PPC‐TX electrolyte and Li metal was significantly improved after the addition of TEGDME to the PPC, even at 30 °C. Furthermore, the cyclability of the Li/PPC‐T5/Li cell was tested at a fixed current density of 0.1 mA cm^−2^ at 30 °C to investigate the long‐term stability with Li metal, as shown in Figure S10b, Supporting Information. The Li/Li symmetric cell with PPC‐T5 exhibited an excellent stability of over 350 h without an increase in *η*. This also confirms the excellent stability of PPC‐T5 with Li metal, even at 30 °C.

### 2.6 Compatibility of PPC‐TX with LiFePO4 (LFP) Cathode

**Figure 5:**
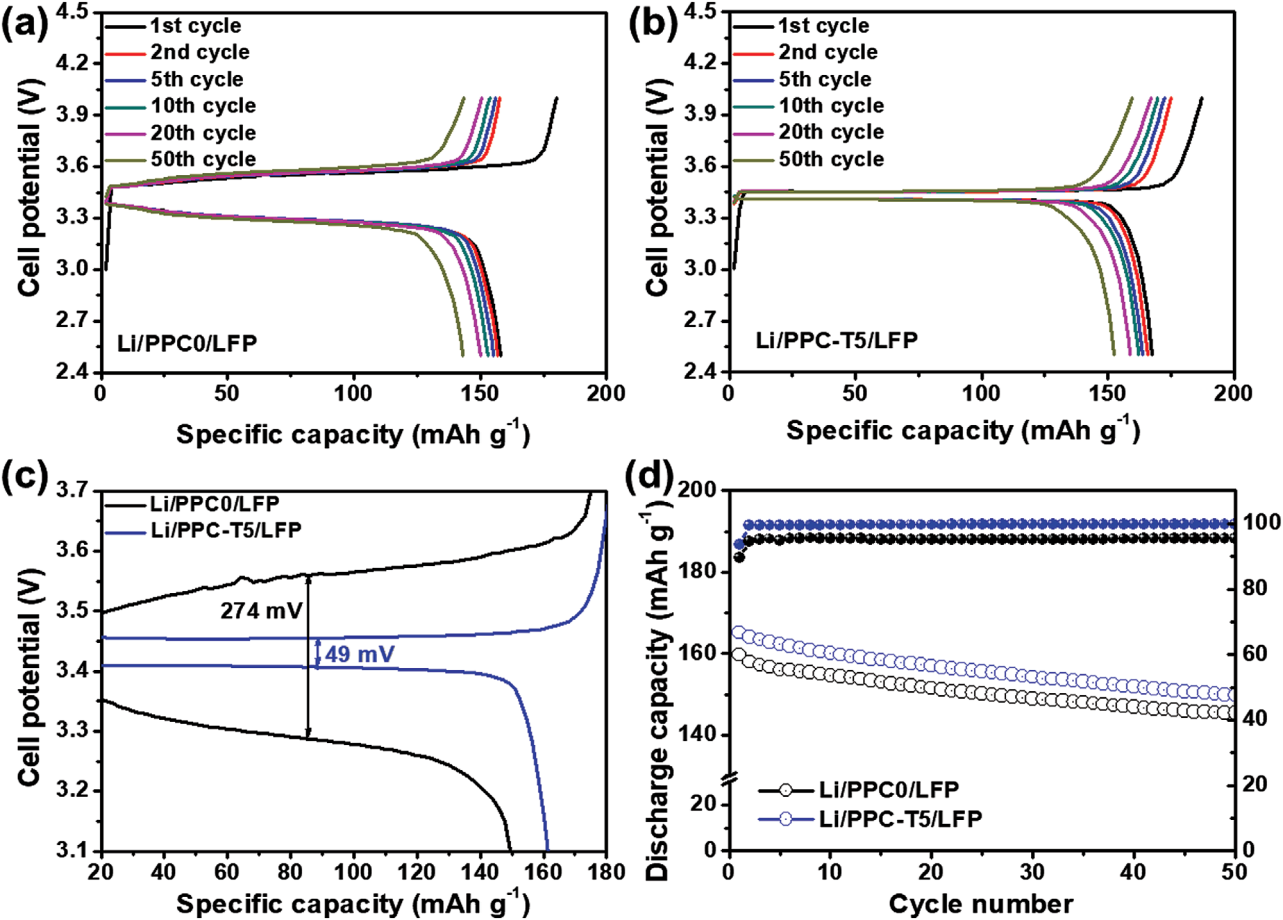
Galvanostatic charge–discharge curves of a) Li/PPC0/LFP and b) Li/PPC‐T5/LFP cells at a rate of 0.1 C at 60 °C. c) Magnified plot of Li/PPC0/LFP and Li/PPC‐T5/LFP cells at a rate of 0.1 C at 60 °C. d) Cyclability of Li/PPC0/LFP and Li/PPC‐T5/LFP cells at a rate of 0.1 C at 60 °C.

The compatibility between fabricated electrolyte PPC‐TX and LFP cathode was understood by conducting galvanostatic charge–discharge tests on the Li/LFP full cells with PPC0 and PPC‐T5 at 60 °C. **Figures** **5**a,b show the galvanostatic charge–discharge profiles of the Li/PPC0/LFP and Li/PPC‐T5/LFP cells at a current of 0.1 C in the cell potential range of 2.5–4.0 V. The Li/PPC0/LFP cell exhibited first cycle charge and discharge capacities of 178 and 160 mAh g^−1^, respectively, corresponding to an initial coulombic efficiency (CE) of 89%, whereas the Li/PPC‐T5/LFP full cell showed the initial charge and discharge capacities of 176 and 165 mAh g^−1^, respectively, corresponding to an initial CE of 94%. The Li/PPC‐T5/LFP full cell had a higher reversible capacity and initial CE than the Li/PPC0/LFP full cell at 0.1 C. Moreover, the potential gap (*η*) between the charge and discharge plateau can be considered as the total overpotential. In the first cycle, the Li/LFP full cells with PPC0 and PPC‐T5 were compared in terms of this potential gap, as presented in Figure 5c. A Li/PPC‐T5/LFP exhibited a considerably smaller *η* of 49 mV, whereas Li/PPC0/LFP cell had a large *η* of 274 mV. The *η* of Li/PPC‐T5/LFP was ≈5.6‐fold less than Li/PPC0/LFP and changed negligibly over the 50 cycles. Figure 5d shows the cyclability of the Li/PPC0/LFP and the Li/PPC‐T5/LFP cells at 0.1 C. The Li/PPC‐T5/LFP full cell exhibited a reversible capacity of 150 mAh g^−1^ over 50 cycles, corresponding to a capacity retention of 91% and a CE of nearly 100%. By contrast, the Li/PPC0/LFP full cell delivered a reversible capacity of 145 mAh g^−1^ over 50 cycles, corresponding to a capacity retention of 90% and a CE of 95%. This indicates that PPC‐T5 provides outstanding stability of PPC‐T5 electrolyte with LFP cathode at a low current.

**Figure 6:**
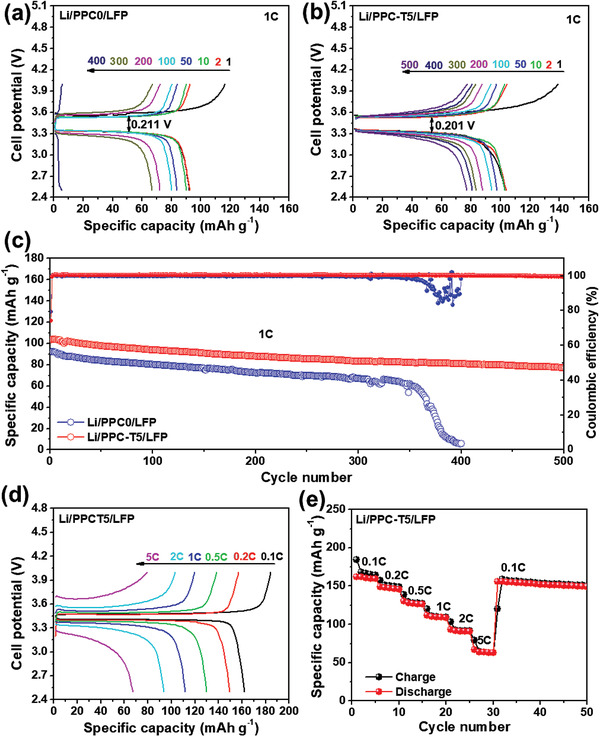
Galvanostatic charge–discharge curves of a) Li/PPC0/LFP and b) Li/PPC‐T5/LFP cells at a rate of 1 C at 60 °C and c) corresponding cyclability. d) Galvanostatic charge–discharge curves of Li/PPC‐T5/LFP cells at different rates and e) corresponding rate capability at 60 °C.

To investigate the long‐term stability of the Li/LFP full cell at a high enough current, the Li/LFP cell with PPC0 and PPC‐T5 electrolytes were tested at a rate of 1 C (or 170 mA g^−1^). **Figures** **6**a,b display the galvanostatic charge–discharge plots of the Li/LFP full cells with PPC0 and PPC‐T5 electrolytes at a rate of 1 C. The Li/PPC0/LFP and Li/PPC‐T5/LFP cells provided initial reversible capacities of 92 and 103 mAh g^−1^, respectively. Moreover, the Li/LFP with PPC‐T5 cell had a smaller *η* value of 0.201 V, at a high enough current of 1 C, and the Li/ LFP with PPC0 cell had a *η* value of 0.211 V. This indicated that the higher ionic conductivity of PPC‐T5 contributed toward reducing *η* throughout the cycling process.

The long term cyclability of the Li/LFP full cell is necessary for durable ASSLIBs. Figure 6c displays the cyclability of the Li/LFP cell with PPC0 and PPC‐T5 electrolytes at a current of 1 C. The Li/PPC‐T5/LFP full cell provided a specific reversible capacity of 79 mAh g^−1^, corresponding to a reversible capacity retention of 77% after 500 cycles. However, the Li/PPC0/LFP full cell deteriorated significantly after 350 cycles. It is possible that the high interfacial resistance between the SPEs and the cathode may lead to a highly unstable interfacial resistance, which can significantly reduce the stability of the battery.^[^ ^43^ ^]^ Moreover, the Li/PPC‐T5/LFP full cell displayed a superior CE of nearly 100% after 500 cycles, whereas the CE of the Li/PPC0/LFP cell degraded after 350 cycles. This indicated that PPC‐T5 provided a higher stability with LFP than PPC0, even at a high rate. As a result, the Li/LFP cell with PPC‐T5 not only offers higher cycling stability, but also superior reversibility for high‐rate charge–discharge cycling. Furthermore, the Li/PPC‐T5/LFP cell's rate capacity was evaluated every 5 cycles at various current ranging from 0.1 C to 5 C, and then maintained at 0.1 C for the next 50 cycles, as shown in Figure 6d,e. The Li/PPC‐T5/LFP cell provided reversible capacities of 162, 149, 130, 112, 93, and 67 mAh g^−1^ at rate of 0.1 C, 0.2 C, 0.5 C, 1 C, 2 C, and 5 C, respectively. In addition, when the rate was reduced to 0.1 C, the Li/PPC‐T5/LFP cell had a reversible capacity of 156 mAh g^−1^ and a capacity retention of 94% after 50 cycles.

### 2.7 Interfacial Analysis of Li Metal

**Figure 7:**
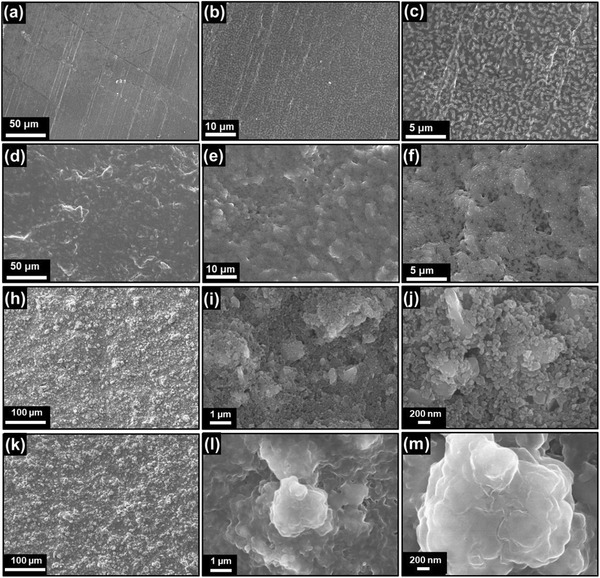
SEM images of a–c) fresh Li metal and d–f) Li electrode from the Li/PPC‐T5/Li cell after 1650 cycles for a current density of 0.1 mA cm^−2^ at 60 °C. Low‐ and high‐magnification SEM images of h–j) pristine LFP electrode and k–m) LFP electrode from the Li/PPC‐T5/LFP cell after 500 cycles for 1 C at 60 °C.

To observe the morphology of the interfacial layer on Li metal, SEM images and XPS data of the Li surface were obtained after 1650 cycles of Li plating/stripping for the Li/PPC‐T5/Li cell at temperature of 60 ℃. The morphologies of the top surface of the Li metal before and after cycling were compared. **Figure** **7**a–c shows low‐ and high‐magnification SEM images of the fresh Li metal, and Figure 7d–f shows the low‐ and high‐magnification SEM images of the Li metal obtained from the Li/PPC‐T5/Li cell after 1650 cycles. A smooth surface was observed for the bare Li metal, whereas a suppressed protrusion‐type byproduct was observed on the surface of the Li metal after cycling with the PPC‐T5 film. Based on this observation, the suppressed protrusions do not appear to penetrate the electrolyte but could instead push the entire electrolyte film forward.^[^ ^42^ ^]^ The suppression of the growth of Li byproduct might be attributed to the high modulus of the PPC‐T5 film, which enabled the long‐term, stable performance of the Li/PPC‐T5/Li cell.^[^ ^35^ ^]^ In addition, the morphology of the byproduct formed on the Li metal was also observed after the Li/PPC‐T5/Li cell was cycled at 30 ℃. Figure S11, Supporting Information, shows the SEM images of the Li metal from the Li/PPC‐T5/Li cell after 350 cycles at 30 ℃. Several lumps were observed on the Li surface, whereas most of the surface of the Li metal was clear without dendrite growth. This also confirmed the formation of protrusions that could not penetrate the PPC‐T5 film owing to the high mechanical strength and good stability of the Li/PPC‐T5/Li cell at 30 ℃, highlighting the potential for the fabrication of cells with high ionic conductivity and high‐modulus SPE structures to prevent dendrite formation and growth.

### 2.8 Interfacial Analysis of LFP Cathode

A detailed understanding of the mechanism and architecture of the cathode electrolyte interface (CEI) is critical for the development of an effective cathode in ASSLIBs. Therefore, we investigated the morphology and chemical composition of the CEI to obtain an in‐depth understanding of the process, which is also relevant to other cathode materials used in ASSLIBs. The morphology and topography of the CEI formed on the LFP electrode surface were analyzed using SEM and AFM, respectively. The various magnified SEM images of a pristine LFP electrode and LFP electrode from the Li/PPC‐T5/LFP cell after 500 cycles are shown in Figure 7k–m. In the pristine LFP electrode, the large particles are associated with the LFP active material, and the small particles belong to the carbon conducting agent (Figure 7h–j). In the case of the LFP electrode, after several cycles with PPC‐T5, a smooth CEI and suppressed, protrusion‐type CEI were observed on the surface of the LFP electrode. This indicated that the suppressed protrusions do not penetrate the electrolyte film.

**Figure 8:**
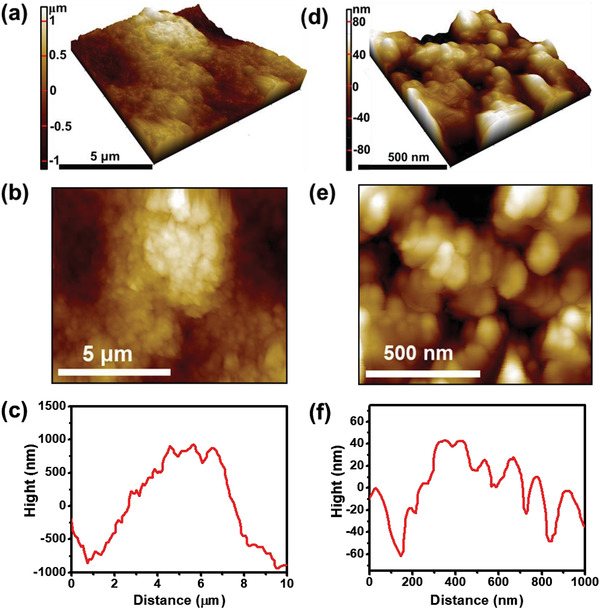
AFM topography images and corresponding line profile of LFP electrode surface at different magnifications after the Li/PPC‐T5/LFP cell was operated for 500 cycles at 1 C.

Furthermore, the topography of the CEI formed on the surface of the LFP electrode obtained from the Li/PPC‐T5/LFP cell tested for 500 cycles was explored in detail using AFM, and **Figure** **8** shows the corresponding line profiles at different magnifications. Figure 8a–c shows the 3D and 2D topographs of an area of 10 × 10 µm^2^ and the corresponding line profile for the LFP electrode. A large lump, which could be ascribed to the suppressed protrusion of the CEI layer, was observed on the LFP electrode. The line profile indicated that the horizontal length and height of the protrusion were ≈7 and ≈1.5 µm, respectively. In addition, Figure 8d–f shows the 3D and 2D topographs of a smaller area of 1 × 1 µm^2^ and the corresponding line profile for the LFP electrode. A protrusion was found to be composed of small granules. This suggests that the protrusion originated from small nanometer‐sized granular structures that coalesced to a size of a few micrometers. The suppressed protrusions on the CEI layer are ascribed to the high modulus of the PPC‐T5 film, which hinted at the long‐term, stable cycle life of the Li/PPC‐T5/LFP full cell.

**Figure 9:**
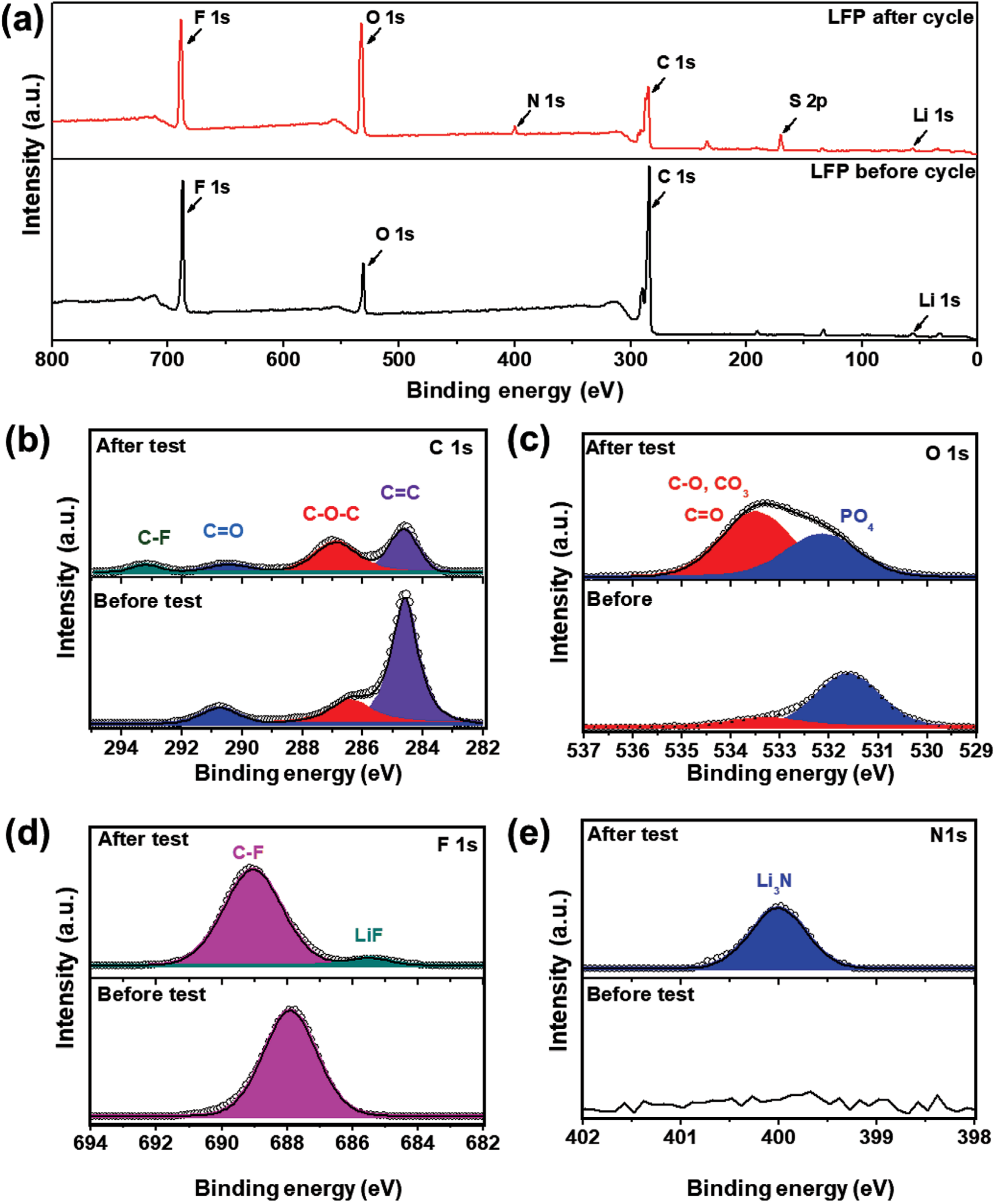
a) Full‐scan XPS spectrum, b) C 1s spectrum, c) O 1s spectrum, d) F 1s spectrum, and e) N 1s spectrum of LFP electrode surface before and after the Li/PPC‐T5/LFP cell was operated for 500 cycles at 1 C and 60 °C.

The chemical composition of the CEI layer on the surface of LFP electrode was examined using XPS and Time‐of flight scanning ion mass spectroscopy (ToF‐SIMS). **Figure** **9**a shows the XPS survey spectra for the Li/PPC‐T5/LFP cell obtained before the test and after 500 cycles at 60 ℃. The full‐scan XPS spectrum of the pristine LFP electrode shows the presence of F, O, C, and Li, whereas that of the LFP electrode after 500 cycles reveals F, O, N, C, S, and Li. Furthermore, high‐resolution, deconvoluted XPS peaks corresponding to C 1s, O 1s, F 1s, and N 1s of the surface of the LFP electrode were compared before and after the cycles (Figure 9b–e). In the C 1s XPS spectra, the pristine LFP electrode showed the presence of C═C (284.6 eV) and C—O—C (286.9 eV) originating from the Super P carbon. The LFP electrode after the cycling test exhibited a minor peak corresponding to C—F (293.2 eV), which was associated with LIF in the CEI layer.^[^ ^48^, ^49^, ^50^ ^]^ It is noted that the intensity of the C═C and C—O—C XPS peaks decreased after cycling owing to the formation of the CEI layer on the surface of LFP electrode. In the case of the O 1s XPS spectra, the intensity of PO_4_ (232.1 eV) from the LFP was slightly reduced, while that of C—O, CO_3_, and C═O (533.5 eV) increased significantly owing to the formation of the CEI layer on the surface of the LFP electrode. Although C—F (689.0 eV) from the PVDF binder was present in pristine LFP, the presence of LiF (685.4 eV) from the CEI layer was confirmed in the F 1s XPS spectra obtained from the surface of the cycled LFP electrode.^[^ ^51^ ^]^ The decomposition of LiTFSI (399.9 eV for Li_3_N) was confirmed in the N 1s XPS peak of the cycled LFP electrode, whereas no N 1s XPS peaks were identified in the pristine LFP electrode.^[^ ^52^, ^53^ ^]^

The quantitative atomic compositions derived from the XPS spectra of the surface of the LFP electrode before and after the cycling test are compared in Table S1, Supporting Information. Overall, the atomic percentage of Li 1s, O 1s, N 1s, and F 1s increased owing to the formation of the CEI layer on the surface of the LFP electrode after 500 cycles. In summary, LiF, Li_3_N, and Li_2_CO_3_ were distinctly identified on the LFP electrode's surface after cycling, which can be attributed to the electrochemical decomposition of inorganic species and partly to the organic group of the PPC. The LiF layer is believed to be a robust electronic insulator, which provides a stable interface and increases the stability of the cathode.^[^ ^54^ ^]^

**Figure 10:**
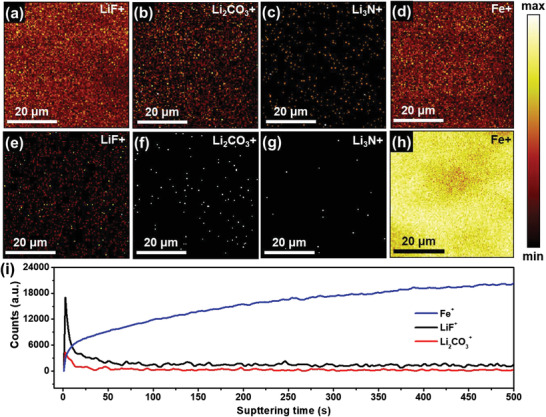
ToF‐SIMS chemical mapping a–d) before and e–h) after sputtering of the LFP electrode; i) depth profile. The LFP electrodes were collected from Li/PPC‐T5/LFP cells operated for 500 cycles at 1 C and 60 °C.

Furthermore, the chemical composition of the outer and inner CEI layer along with the depth distribution in the CEI on the LFP cathode were examined using ToF‐SIMS. **Figure** **10**a–d shows the ToF‐SIMS high‐resolution chemical mapping of LiF^+^, Li_2_CO_3_^+^, Li_3_N^+^, and Fe^+^ species on the LFP electrode's surface after 500 cycles of the Li/PPC‐T5/LFP. In particular, the signal from LiF^+^ was considerably stronger than those from Li_2_CO_3_^+^ and Li_3_N^+^. This indicated that the high LiF^+^ content was uniformly distributed on the surface, which is attributed to the Li salt (LiTFSI) in the electrolyte (PPC‐T5). LiF was shown to play a significant role in building a stable and efficient CEI.^[^ ^53^, ^54^, ^55^, ^56^, ^57^ ^]^ Likewise, the distribution of species in the CEI layer was demonstrated by examining the depth profile produced on sputtering the sample for 500 s. In Figure 10i, the signal from the Fe^+^ species (due to the LFP cathode) increased with time. However, the signal from the LiF^+^ and Li_2_CO_3_^+^ species decreased with increasing sputtering time. Remarkably, the initial intensity of the LiF^+^ species was significantly higher than that of Li_2_CO_3_^+^, and the saturation of the LiF^+^ species was mostly stable for a few seconds throughout the sputtering process. In addition, we investigated the chemical mapping of LiF^+^, Li_2_CO_3_^+^, Li_3_N^+^, and Fe^+^ species after sputtering for 500 s to understand the chemical composition of CEI near the LFP cathode (Figure 10e–h). The signal and distribution of LiF^+^ were significantly greater than those of Li_2_CO_3_^+^ and Li_3_N^+^ even after sputtering. This indicated that the LiF^+^ species was uniformly distributed throughout the CEI layer. LiF has been shown to play a critically important role in the formation of a stable and efficient CEI.^[^ ^53^ ^‐57]^ TEGDME, a commonly used ether solvent with dielectric constant (7.9) and high donor number (18.6), can dissociate large amounts of lithium salts with weak solvating ability around Li^+^ cations. Therefore, the participation of TFSI^−^ anions in the solvation structure is increased and decomposition is accelerated at the electrode interface. This leads to high LiF content in CEI during electrochemical process. Consisting of a stable LiF‐rich layer, CEI effectively protects the LFP electrode. Accordingly, this layer can improve the overall electrochemical performance of PPC‐T5 using LFP cathode.

## 3 Conclusion

We demonstrated the effect of the TEGDME additive in PPC‐based SPEs. The PPC‐T5 SPE with the optimal TEGDME concentration of 5 wt% exhibited a high ionic conductivity of 0.89 mS cm^−1^ and Li‐ion transference number of 0.81 at 60 ℃. In particular, the mechanical strength of the SPE was maintained by employing a tissue membrane as a backbone. The tensile strength of PPC‐T5 was ≈6.9 MPa, which was almost 2.2‐fold higher than that of the pure PPC film. The micro‐ and nano‐pores of the tissue membrane increased the surface area for interaction and thus improved the overall mechanical strength of the SPE. The chemical interaction and Li‐ion coordination between PPC and TEGDME were also examined using XPS and solid‐state MAS‐NMR analyses. Additionally, DFT calculations were performed to investigate the effect of the additive on the electrochemical potential stability of the SPE. According to the DFT results, the HOMO energy of TEGDME was −5.79 eV, which was higher than that of LiTFSI (−7.26 eV) and PPC (−6.60 eV). Thus, the addition of the TEGDME additive to the PPC polymer lowered the anodic oxidation potential. Furthermore, the interfacial contact of the SPEs with Li metal and the LFP electrode was significantly improved after the addition of TEGDME to the PPC polymer because the surface of the SPE was softened. The Li/PPC‐T5/Li symmetric cell showed an excellent stability of up to 1650 h. The Li/PPC‐T5/LFP cell had a high initial reversible capacity of 103 mAh g^−1^ and a capacity retention of 77% after 500 cycles under a current of 1 C at 60 ℃. Moreover, the surface morphology and topography of the interface formed on the Li metal and LFP electrode were examined using SEM and AFM. A large lump was observed on the LFP electrode, which could be ascribed to the suppressed protrusion of the CEI. This protrusion originated from the small, granular, and nanometer‐sized structures that coalesce to produce a few micrometer‐sized structures. Furthermore, the chemical composition of the CEI on the LFP electrode surface was examined using XPS and ToF‐SIMS. LiF, LiTFSI, and Li_2_CO_3_ were confirmed to be present on the LFP electrode surface after cycling, which can be primarily attributed to the electrochemical decomposition of inorganic species and partly to the organic group of the PPC. The LiF layer is believed to be a robust electronic insulator that provides a stable interface and increases the stability of the cathode. The signal from LiF^+^ in the ToF‐SIMS chemical mapping was considerably stronger than those from Li_2_CO_3_^+^ and Li_3_N^+^, even after sputtering for 500 s. This indicated that the LiF^+^ species was uniformly distributed throughout the CEI layer. It was demonstrated that LiF played a critically important role in the fabrication of a CEI with a stable and efficient LiF‐rich layer to effectively protect the LFP electrode. Therefore, this CEI layer could enhance the overall electrochemical performance of ASSLIBs. Our work could facilitate the development of simple, cost‐effective ASSLIBs with excellent electrochemical performance.

## 4 Experimental Section

### Preparation of Electrolyte

The fabrication process of the SPE film is shown in Scheme 1. PPC and LiTFSI were added in 8 mL of acetonitrile while constantly stirring overnight, followed by the addition of TEGDME. The solution was sonicated and constantly stirred for 8 h at 30 ℃. The homogenous solution of the electrolyte was poured onto a tissue membrane and dried for 48 h at 30 ℃ under an Ar atmosphere and then for 12 h at 80 ℃ under vacuum to evaporate the moisture. The TEGDME content in the electrolyte was altered as 0, 2, 5, and 8 wt% with respect to PPC(LiTFSI), denoted as PPC0, PPC‐T2, PPC‐T5, and PPC‐T8, respectively. The thickness of the SPE films was maintained at ≈150 ± 5 µm.

### Preparation of LiFePO_4_ (LFP) Electrode

The LiFePO_4_ (LFP) electrode was fabricated using the following ratio: LFP:Super P:polyvinylidene fluoride (PVDF):PPC(LiTFSI) = 70:20:5:5. The homogeneous slurry of the LFP, Super P, PVDF, and PPC(LiTFSI) was casted onto an aluminum current collector. The fabricated electrodes were kept at 80 °C in air for 30 min, followed by heating at 90 °C in a vacuum for 24 h to fully vaporize the *N*‐methyl‐2‐pyrrolidone (NMP) solvent. The diameter and mass of the LPF electrodes were maintained as 1.4 cm and 3.6 mg cm^−2^, respectively.

### Material Characterization

The tops and cross‐sections of the SPEs, Li metal, and LFP electrodes were observed using SEM (S‐4700/EX‐200; Hitachi, Japan), and the corresponding elemental mapping was obtained using energy‐dispersive X‐ray spectroscopy (EDS). The phase of the SPEs was confirmed using high‐resolution X‐ray diffraction (HR‐XRD; Cu K_*α*_, 2 kW, EMPyrean, PANalytical). Thermogravimetric analysis (TGA‐50, Shimadzu Japan) performed to inspect the thermal stability of the SPEs, and their glass transition temperature (*T_g_*) was evaluated using differential scanning calorimetry (DSC, Shimadzu Japan). The XPS (VG Multilab 2000) and Fourier‐transform infrared spectroscopy (FTIR; Spectrum 400) analyses were carried out to study the interaction between PPC and TEGDME. Li coordination with and without TEGDME was investigated using ^7^Li magic‐angle‐spinning (MAS) solid‐state NMR spectroscopy on a Bruker instrument. Universal Testing (Koptri, Korea) Machine was used to check the mechanical strength of SPEs with and without TEGDME at a pulling rate of 0.1 mm min^−1^.

### Electrochemical Characterization

Electrochemical characterization was performed after the fabrication of a coin cell (CR2032) in an Ar‐filled glovebox. EIS (Zive Lab SP2, 10 mV, 2 A) of the SS (stainless steel)/SPEs/SS cell was performed to determine the total ionic conductivity (*σ*) of the SPEs by Equation 1:

(1) σ=t/RA

where *t*, *A*, and *R* are the thickness, area, and resistance of the SPEs. A LSV (Gamry‐PC 750 potentiostat) of the Li/SPEs/SS symmetric cell was obtained to examine the voltage stability. Furthermore, the Li‐ion transference number (*t*_Li+_) and the stability of the SPE with Li metal were examined by assembling a Li/SPEs/Li cell. A single‐step chronoamperometry method (Zive Lab SP2) with an overpotential (*∆V*) of 10 mV was utilized, and *t*_Li+_ was calculated using Equation (2):

(2) $$t_{\mathrm{Li}}^{+}=\frac{I_{\mathrm{ss}}\left(\Delta V-I_{0}R_{0}\right)}{I_{0}\left(\Delta V-I_{\mathrm{ss}}R_{\mathrm{ss}}\right)}$$

where, *I*_o_ = initial current, *I*_ss_ *=* steady‐state currents, *R*_o_ = resistance before *∆V*, and *R*_ss_ = resistance after *∆V*. A battery cycler with multichannel (WonATech/WBCS 3000) was used to perform galvanostatic charge/discharge of the Li/Li cell and the Li/LFP full cell with SPEs in order to investigate the compatibility of the SPEs with Li metal and the LFP electrode, respectively.

### Computational Method

The implemented computational methods were based on DFT with the Quantum Espresso package using an ultra‐soft pseudo‐potential, the generalized gradient approximation in the Perdew–Burke–Ernzerhof (PBE) exchange‐correlation functional in the Pslibrary.^[^ ^60^, ^61^, ^62^, ^63^ ^]^ The atoms in all the structures were relaxed using the Broyden–Fletcher–Goldfarb–Shanno (BFGS) algorithm with force and energy convergences of less than 10^−5^ Ry/Bohr and 10^−5^ Ry, respectively. Both the cut‐off energy and the k‐point sampling were optimized by using a self‐consistent field and set to 50 Ry using a 3 × 3 × 3 grid. VESTA was used for visualization.^[^ ^64^ ^]^

### Interface Characterization

The morphology of the solid electrolyte interface (SEI) between SPEs and the Li metal was observed via SEM after 1650 h of the Li/SPEs/Li cell. Moreover, the morphology and topography of the cathode–electrolyte interface (CEI) between SPE and LFP were investigated using SEM and AFM (XE‐100, non‐contact mode) for different surface areas of the LFP electrode after 500 cycles of the Li/SPEs/Li cell. Furthermore, the chemical composition of the CEI and its distribution on the LFP electrode were investigated using XPS and ToF‐SIMS (ION‐TOF GmbH, Munster, Germany). The ToF‐SIMS depth profile was obtained using the positive mode (O_2_, 2 keV) with an etching area of 500 × 500 µm^2^.

## Conflict of Interest

The authors declare no conflict of interest.

## Supporting information

Supporting Information

## Data Availability

Research data are not shared.
