# Supplementary material for: Improving Cyclability of All‐Solid‐State Batteries via Stabilized Electrolyte–Electrode Interface with Additive in Poly(propylene carbonate) Based Solid Electrolyte
Source: Adv Sci (Weinh). 2022 Mar 3;9(13):2105448. doi: 10.1002/advs.202105448 (PMC9069196; doi:10.1002/advs.202105448)
Supplement: Supplementary file 1 — Supporting Information [file ADVS-9-2105448-s001.pdf]

ADVANCED SCIENCE
Open Access

Supporting Information

for *Adv. Sci.*, DOI 10.1002/advs.202105448

Improving Cyclability of All-Solid-State Batteries via Stabilized Electrolyte–Electrode Interface with Additive in Poly(propylene carbonate) Based Solid Electrolyte

*Pravin N. Didwal, Rakesh Verma, An-Giang Nguyen, H. V. Ramasamy, Gwi-Hak Lee and Chan-Jin Park**

Supporting information

Improving Cyclability of All-Solid-State Batteries via Stabilised Electrolyte–Electrode Interface with Additive in Poly(Propylene Carbonate) Based Solid Electrolyte

*Pravin N. Didwal, Rakesh Verma, An-Giang Nguyen, H. V. Ramasamy, Gwi-Hak Lee, Chan-Jin Park **

Table S1. Quantitative atomic composition from the XPS spectra of the LFP electrode surface before and after 500 cycles

Element	Atomic %	
	Pristine LFP electrode	LFP electrode after 500 cycles
Li 1s	14.47	16.87
C 1s	61.91	31.89
N 1s	0.25	2.08
O 1s	7.24	26.23
F 1s	16.11	19.26
S 2p	0.03	3.68

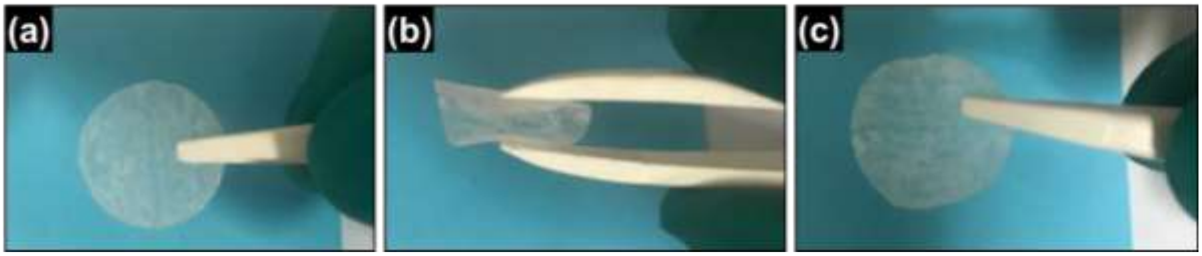

Figure S1. Photographs of a typical PPC-T5: (a) fresh PPC-T5, (b) twisted PPC-T5, and (c) after twisting.

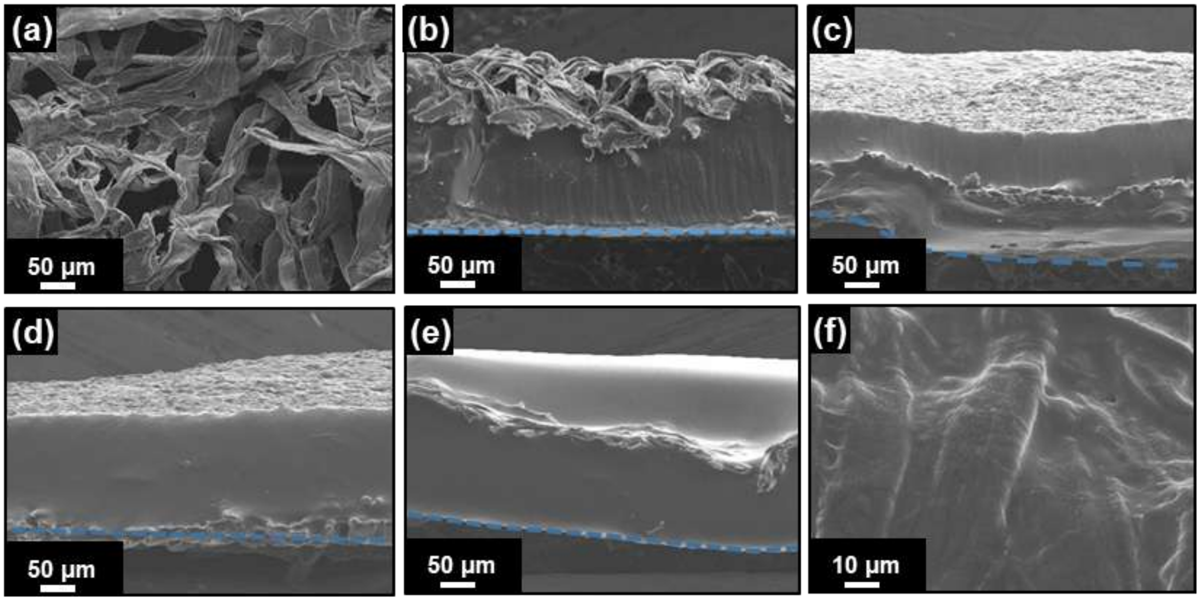

Figure S2. (a) Top-surface SEM images of tissue paper at low magnification. Side-view SEM images of (b) PPC0, (c) PPC-T2, (d) PPC-T5, and (e) PPC-T8. (f) Top-view SEM image of PPC0.

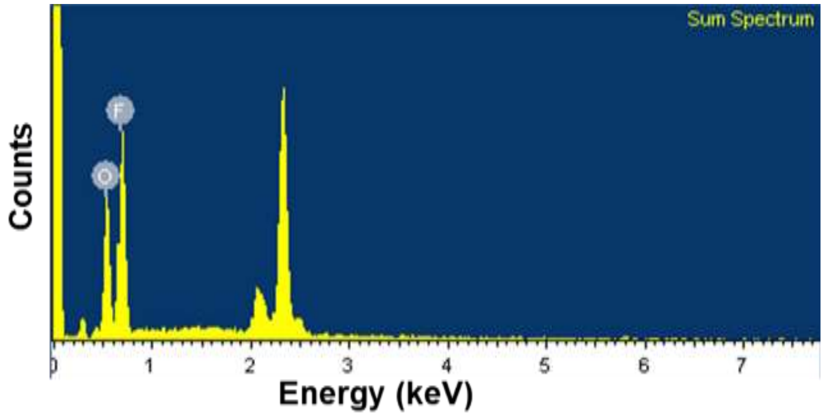

Figure S3. SEM-EDS spectrum of PPC-T5 corresponding to the cross-sectional SEM image shown in Figure 1g.

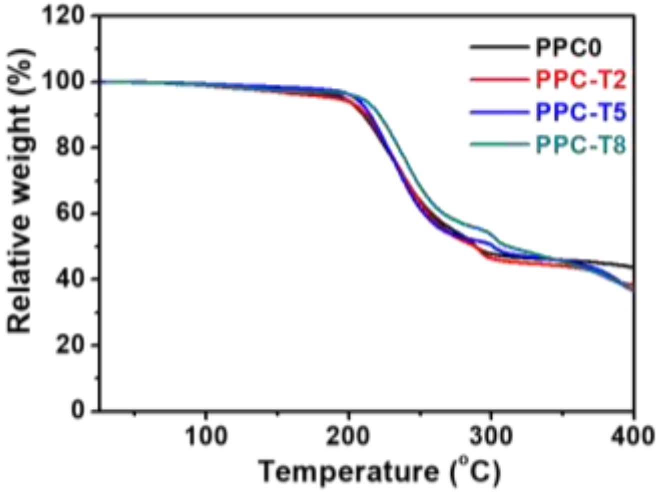

Figure S4. TGA spectra of PPC0, PPC-T2, PPC-T5, and PPC-T8.

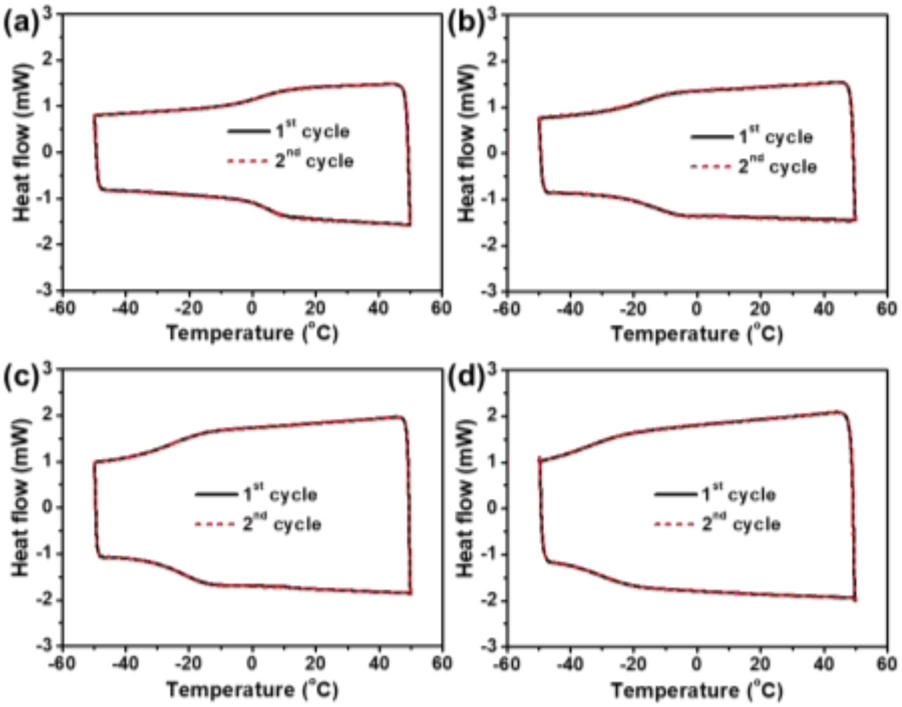

Figure S5. DSC curves of (a) PPC0, (b) PPC-T2, (c) PPC-T5, and (d) PPC-T8.

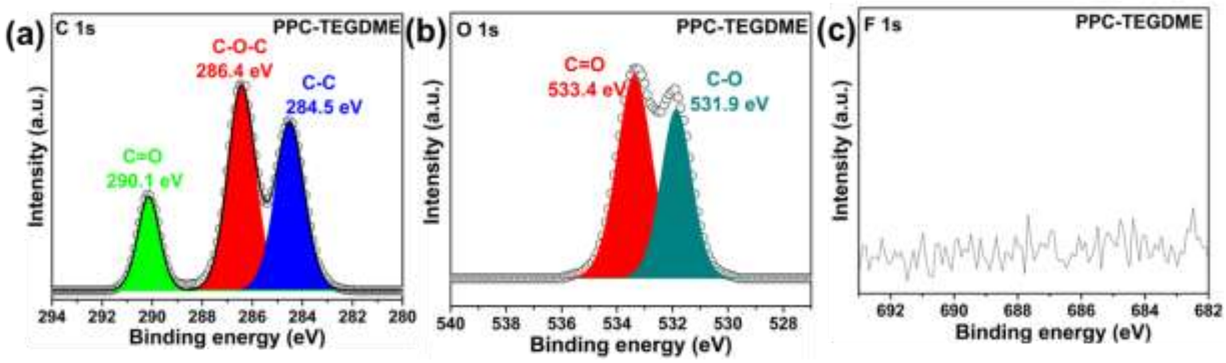

Figure S6. (a) C 1s, (b) O 1s, and (c) F 1s core XPS spectra of PPC+TEGDME.

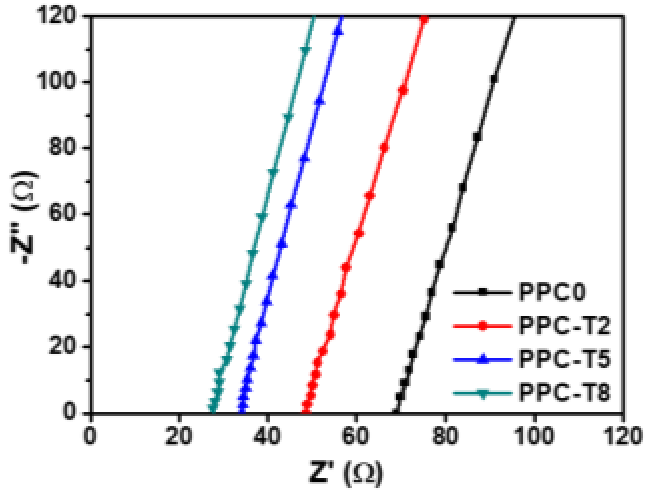

Figure S7. Nyquist plot of PPC0, PPC-T2, PPC-T5, and PPC-T8 at 60 °C.

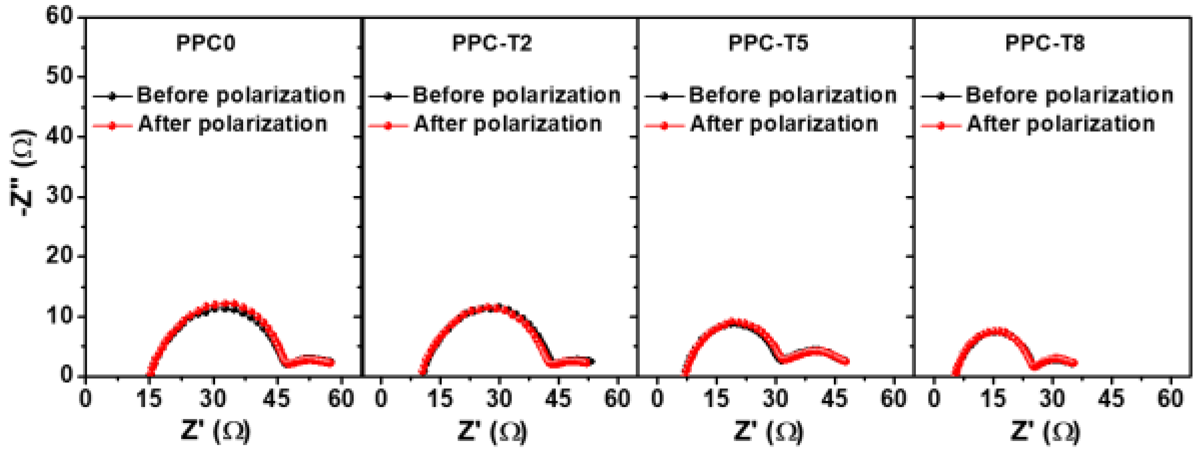

Figure S8. Nyquist plots of Li/Li symmetric cell before and after polarisation at 60 °C.

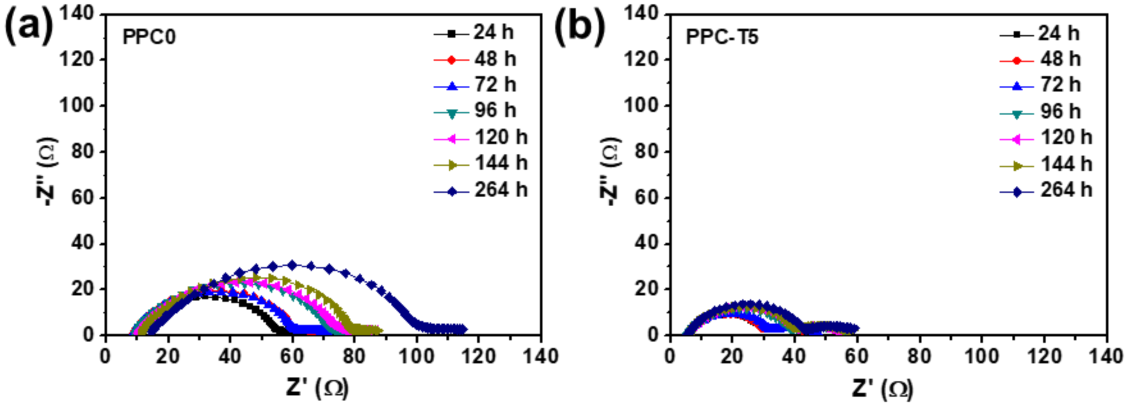

Figure S9. Nyquist plot of Li/Li symmetric cell with (a) PPC0 and (b) PPC-T5 as a function of the aging time.

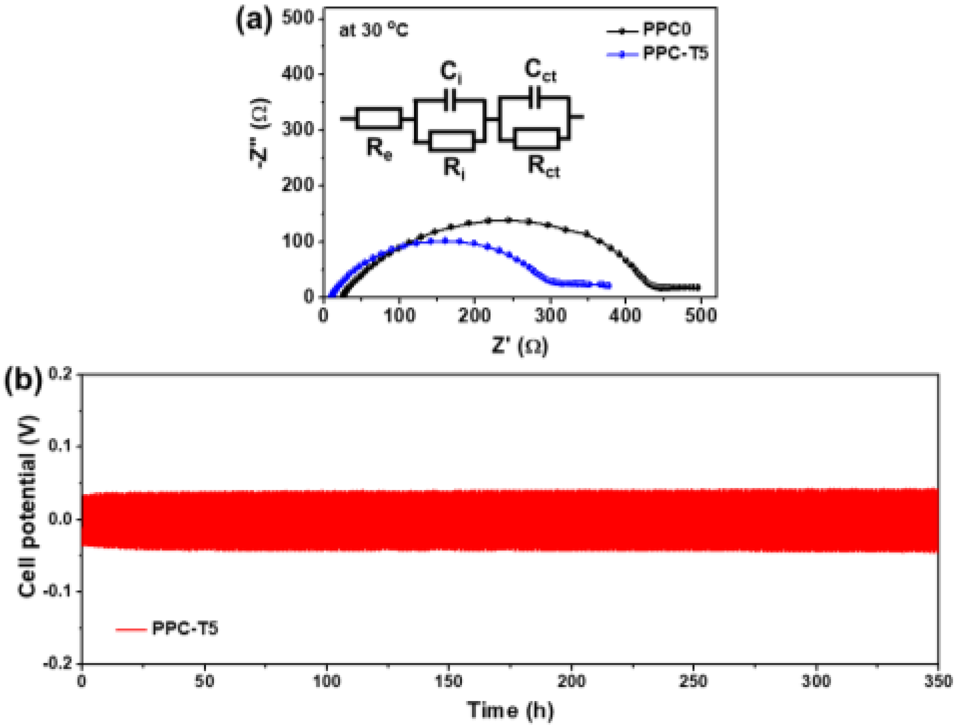

Figure S10. (a) Nyquist plot of Li/Li symmetric cells with PPC0 and PPC-T5 at 30 °C. (b) Cyclability of an Li/PPC-T5/Li symmetric cell at a current density of 0.1 mA cm^{-2} and 30 °C.

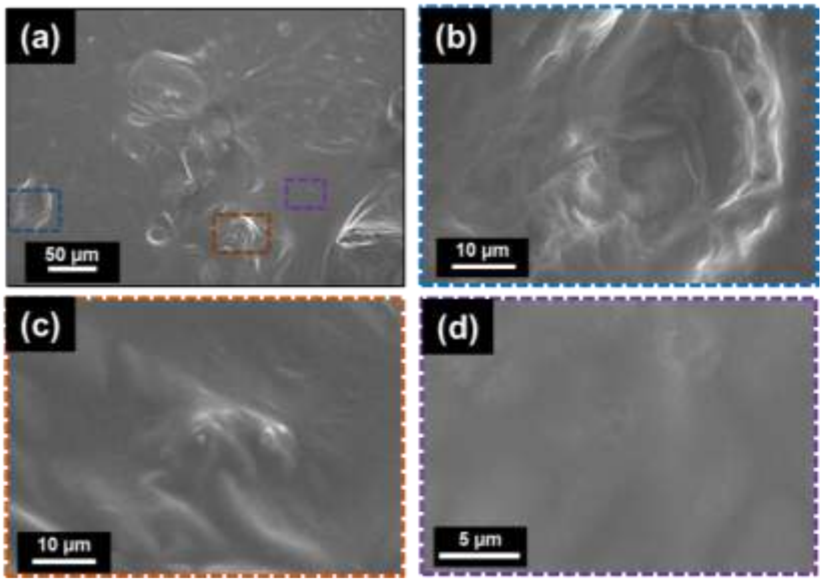

Figure S11. High- and low-magnification SEM images of Li electrode from the Li/PPC-T5/Li cell after cyclic testing for 350 cycles with a current density of 0.1 mA cm^{-2} at 30 °C.

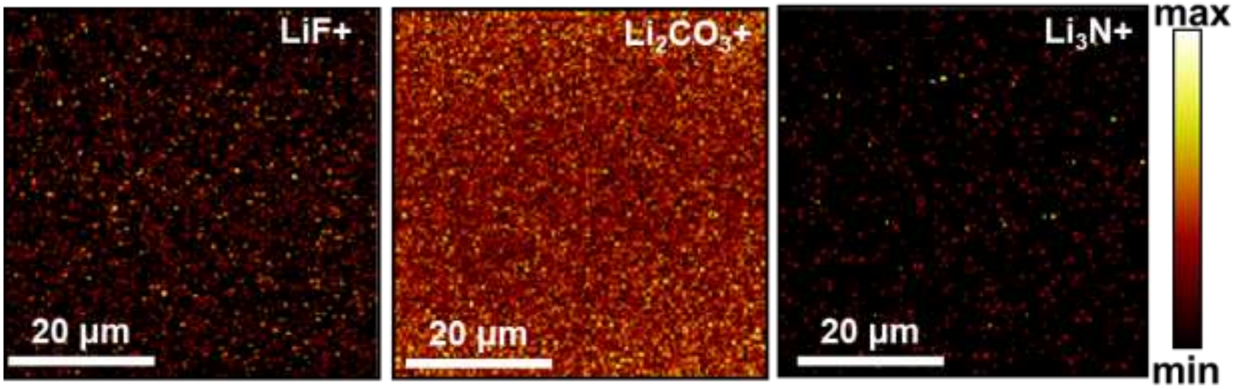

Figure S12. ToF-SIMS chemical mapping of LFP electrode after the Li/PPC-T5/LFP cell was operated for 500 cycles at 1 C and 60 °C.

ToF-SIMS was used to investigate the chemical composition of the CEI layer on the LFP cathode facing the PPC0 electrolyte film. **Figure S11** shows the ToF-SIMS high-resolution chemical mapping of LiF^+, $Li_2CO_3^+$ and Li_3N^+ species present on the LFP electrode surface after 500 cycles of Li/PPC0/LFP. In particular, the signal of LiF^+ was significantly weaker than that of $Li_2CO_3^+$ and similar to that of Li_3N^+. This indicated that PPC0 film (without TEGDME) produced the less LiF^+ content than PPC-T5 film (with TEGDME). Thus, the participation of $TFSI^-$ anions is less in the solvated structure and leads to lower LiF content in CEI during electrochemical processing. [S1]

References.

[S1]. O. Sheng, J. Zheng, Z. Ju, C. Jin, Y. Wang, M. Chen, J. Nai, T. Liu, W. Zhang, Y. Liu, X. Tao, Adv.Mater., 2020, 32, 2000223.
